# The Variation Tendency of Polyamines Forms and Components of Polyamine Metabolism in Zoysiagrass (*Zoysia japonica* Steud.) to Salt Stress with Exogenous Spermidine Application

**DOI:** 10.3389/fphys.2017.00208

**Published:** 2017-04-06

**Authors:** Shucheng Li, Linlin Cui, Yujuan Zhang, Yunwen Wang, Peisheng Mao

**Affiliations:** ^1^Department of Grassland Science, China Agricultural UniversityBeijing, China; ^2^Institute of Grassland Science, Chinese Academy of Agricultural ScienceHuhhot, China

**Keywords:** zoysiagrass, components, polyamine metabolism, dynamic variation, exogenous spermidine, salinity stress

## Abstract

To understand dynamic changes in polyamines (PAs) forms and components of polyamine metabolism in zoysiagrass (*Zoysia japonica* Steud.) response to salt stress with exogenous spermidine (Spd) application, two Chinese zoysia cultivars, z081 and z057, were exposed to sodium chloride stress for 2, 4, 6, and 8 days. The z057 cultivar possesses higher salinity tolerance than the z081 cultivar. Salt stress decreased the zoysiagrass fresh weight (FW) and increased free Spd and spermine (Spm) levels and soluble and insoluble putrescine (Put), Spd and Spm levels in both cultivars. Moreover, salt stress enhanced the activities of arginine decarboxylase (ADC), ornithine decarboxylase (ODC), S-adenosylmethionine decarboxylase (SAMDC), and diamine oxidase (DAO). Exogenous Spd increased PA metabolism and ADC, SAMDC, and DAO activities and decreased free Put levels under salt stress conditions in both cultivars. In addition, structural equation modeling (SEM) showed that ODC, SAMDC, and DAO contributed to PA metabolism, and endogenous Spd levels also contributed to endogenous Spm levels. Free PAs may be the primary factor influencing the variation of other PA forms. SEM also indicated that ADC and polyamine oxidase (PAO) play a limited role in enhancing zoysia salt tolerance via PA metabolism under salt stress.

## Introduction

Salinity stress is a major factor limiting plant growth and restricting the production of high-quality plants. Potential and actual plant yields differ considerably under salt stress. Salinity stress may cause a greater than 50% reduction in major perennial and annual crops worldwide (Wang et al., 2003). Plants have evolved highly coordinated and complex systems to adapt to salt stress using a variety of physiological and biochemical responses. A range of physiological, biochemical, morphological and molecular changes occur in response to salinity stress. Richards and Coleman (1952) determined that polyamine (PA) metabolism is involved in protecting and maintaining the structure and function of cellular components under salt stress. Many studies have implicated PAs in plant growth and development (Pál et al., 2015).

PAs are low-molecular-weight aliphatic cations that are widely present among organisms (Hussain et al., 2011). In plants, PAs are mainly present as three types: the diamine putrescine (Put), the triamine spermidine (Spd), and the tetraamine spermine (Spm). All three major PAs are present in freely soluble forms or bound insoluble forms. The PA biosynthetic pathway bas been extensively studied in plants (Kusano et al., 2008; Vera-Sirera et al., 2010; Pegg and Casero, 2011; Gupta et al., 2013). In plants, ornithine decarboxylase (ODC) and arginine decarboxylase (ADC) catalyze Put production in three steps. Spd synthase (SPDS) catalyzes the formation of Spd from Put and an aminopropy1 moiety donated from decarboxylated S-adenosylmethionine (dcSAM). Spd or thermospermine is synthesized from Spm by Spm synthase (SPMS) using dcSAM as an aminopropyl donor. In addition to the *de novo* synthesis of PAs, PA catabolism involves two classes of enzymes: diamine oxidases (DAOs) and FAD-containing polyamine oxidases (PAOs) (Cona et al., 2006; Alcázar et al., 2010; Moschou et al., 2012).

PAs are involved in various processes in plant growth and development, such as biofilm formation (Lee et al., 2009), xylem differentiation (Tisi et al., 2011), fruit ripening (Gil-Amado and Gomez-Jimenez, 2012), programmed cell death (Kim et al., 2013), and embryogenic competence (Silveira et al., 2013). PAs are involved in salt tolerance, as evidenced by changes in PA concentrations in response to salt stress. The three major PAs (Put, Spd, and Spm) increase in abundance under salt stress (Yang et al., 2007). However, in most cases, only one of the three PAs increases significantly under salt stress (Liu et al., 2006). Wang and Liu (2009) reported that the Spd content increased significantly in response to salt stress, and Ikbal et al. (2014) observed the accumulation of Spd and Spm in response to salt stress. Similar results were observed in 18 rice species under drought stress (Do et al., 2014). In most cases, PA accumulation is higher in tolerant genotypes than in sensitive genotypes (Hatmi et al., 2015). The increased *de novo* synthesis of free PAs is primarily responsible for PA accumulation under salt stress. To understand the regulation of PAs at the transcriptional level, many studies have evaluated steady-state transcript levels of PA biosynthetic genes. The expression of PA biosynthetic genes such as ADC, SPDS, SPMS and SAMDC is increased upon exposure to salt stress (Urano et al., 2004; Liu et al., 2006; Wang et al., 2011; Majumdar et al., 2013; Guo et al., 2014). These studies suggest that the accumulation of PAs is an adaptive mechanism in response to salt stress and that PA dynamics is complex.

The dynamic variation of PA content is considered a response to salt stress. To better understand the roles of PAs in the response to salinity stress, three approaches, including the application of exogenous PAs and PA synthesis inhibitors and the overexpression of biosynthetic genes, have been used. Exogenous Spd application has been shown to enhance the salt tolerance of different plants (Duan et al., 2008). A recent study by Li et al. (2016) demonstrated that application of 0.15 mM Spd alleviated the damage caused by salt stress in zoysia (*Zoysia japonica* Steud.). Exogenous Spd application also enhanced the salt tolerance of sorghum (*Sorghum bicolor*) seedlings (Yin et al., 2016). Hu et al. (2012) also found that exogenous application of 0.15 mM Spm reduced salt injury under salt stress in tomato. Several transgenic techniques for overexpressing genes encoding PA biosynthetic enzymes have been widely applied in rice and *Arabidopsis thaliana* (Roy and Wu, 2001; Kasukabe et al., 2004, 2006).

There is substantial evidence that PA (Put, Spd, Spm) levels undergo extensive changes under salinity stress with exogenous Spd application. However, these PAs (Put, Spd, Spm) are present in three forms (freely soluble, insoluble bound forms, and soluble forms). There have been few reports to date on dynamic variations in different forms of PA to salt stress with exogenous Spd application and components of polyamine metabolism. Our objective was to characterize the forms (freely soluble, insoluble bound forms, soluble forms) of PAs present under the salt stress and the components of PA metabolism in zoysiagrass.

## Materials and methods

### Plant materials and treatments

In this study, two zoysia (*Zoysia japonica* Steud.) cultivars (z057 and z081) were used. z057 is tolerant to salinity stress, whereas z081 is comparatively sensitive to salinity stress (Li et al., 2012). The cultivars were collected from China (Table 1) and cultivated under hydroponic conditions with 1/2 Hoagland solution (pH 6.6 ± 0.1, EC 1.8–2.0 dsm^−1^) with pump aeration (China Agricultural University, Haidian, Beijing, China). The air temperatures at day and night in the greenhouse were 17–20°C and 25–28°C, respectively. The air relative humidity in the greenhouse was 60–70%.

**Table 1:** *****Zoysia japonica*** cultivars used in the study, growth conditions and plant sources**.

**Cultivar**	**Species**	**Source sponsor**	**Source location**
z081	*Z. japonica*	Qingdao, Shandong	36°05′N, 120°20′E
z057	*Z. japonica*	HuaguoShan, Lianyungang	34°36′N, 119°12′E

Zoysia roots were pruned to 5 cm before treatment. Four treatments were then applied: (1) control, consisting of 1/2 Hoagland solution alone; (2) 1/2 Hoagland solution + 0.15 mM Spd; (3) 1/2 Hoagland solution + 150 mM NaCl; and (4) 1/2 Hoagland solution + 150 mM NaCl + 0.15 mM Spd.

Root samples were collected with three replicates on days 0, 2, 4, 6, and 8 after salinity treatment.

### Root growth

The dry weight was determined after drying at 75°C for 72 h.

### PA analysis

The PA extraction method is based on Sharma and Rajam (1995), with some modifications. Cold perchloric acid (PCA, 4 mL, 5% v/v) was added to the fresh root homogenates and incubated for 1 h at 4°C. 1,6-Hexanediamine was added to the homogenates as an internal standard. The homogenates were centrifuged at 12,000 × g at 4°C for 30 min. The supernatants were used to determine free and soluble conjugated PAs, and the residue was used to determine insoluble bound PAs. To determine soluble conjugated PAs, the PCA extract (1 mL) was mixed with 5 mL of 6 N HCl and hydrolyzed at 110°C for 18 h in a flame-sealed glass ampule. After acid hydrolysis, the HCl was evaporated at 70°C, and the residue was suspended in 2 mL of 5% PCA after centrifugation at 12,000 × g for 30 min at 4°C. The solution contained acid-soluble PAs, including those liberated from PA conjugates and free PAs. To determine insoluble bound PAs, the pellets were rinsed four times with 5% PCA to remove any traces of soluble PAs, followed by suspension in 5 mL of 6 N HCl. The same procedure above was used to hydrolyze this solution.

PAs recovered from the hydrolyzed supernatants, nonhydrolyzed supernatants and pellets were benzoylated as follows. An aliquot of supernatant mixed with 2 mL of 2 N NaOH and 15 μL of benzoyl chloride was vortexed vigorously and incubated for 30 min at 37°C. Then, the reaction was terminated via the addition of 4 mL of saturated NaCl solution. Finally, 1.5 mL of the ether phase was dried and redissolved in 1 mL of methanol.

PAs were assayed by high-performance liquid chromatography (HPLC). Ten microliters of methanol solution containing benzoylated PAs was injected into a 20-mL loop and loaded onto a 5-μm particle-size C18 reverse-phase, 4.6-mm × 250-mm column (Eka Chemicals, Bohus, Sweden). The column temperature was maintained at 25°C. Samples were eluted with 64% methanol, and a flow rate of 0.8 mL min^−1^ was maintained using a Dionex P680 Pump. PA peaks were detected with a UV detector at 254 nm. The concentrations of soluble conjugated PAs were calculated by subtracting free PA concentrations from acid-soluble PA concentrations.

### Analysis of PA biosynthetic enzyme activity

Fresh samples were homogenized in 100 mM potassium phosphate buffer (pH 8.0) containing 0.1 mM phenylmethylsulfonyl fluoride, 1 mM pyridoxal phosphate (PLP), 5 mM EDTA, 25 mM ascorbic acid and 0.1% polyvinylpyrrolidone. After centrifugation at 12,000 × g for 40 min at 4°C, the supernatants were dialyzed at 4°C against 3 mL of 100 mM potassium phosphate buffer (pH 8.0) containing 1 mM pyridoxal phosphate (PLP), 0.05 mM PLP, 0.1 mM DTT, and 0.1 mM EDTA for 24 h in the dark. The dialyzed extracts were used for enzymatic assays.

The activities of ODC, ADC, and SAMDC were determined according to the procedure described by Zhao et al. (2003), with some modifications. The reaction mixtures were activated after adding 0.3 mL of the dialyzed enzyme extract and 100 mm Tris-HCl buffer (pH 8.0), 50 μM pyridoxal phosphate, 5 mM EDTA, and 5 mM DTT. Then, the reactions were incubated at 37°C for 2 min, followed by the addition of 0.2 mL of 25 mM L-ornithine, 0.2 mL of 25 mM L-arginine (pH 7.5) or 0.2 mL of 25 mM SAM. Then, the reaction mixtures were incubated at 37°C for 30 min, followed by the addition of PCA to a final concentration of 5%. Reaction mixtures were centrifuged at 3,000 × g for 10 min, and the supernatants (0.5 mL) were mixed with 1 mL of 2 mM NaOH and 10 μL of benzoyl chloride. The mixtures were stirred for 20 s. After incubation at 37°C for 30 min, 2 mL of NaCl solution and 3 mL of ether were added and stirred thoroughly, followed by centrifugation at 1,500 × g for 5 min and extraction with 3.0 mL of ether. Then, 1.5 mL of the ether phase was evaporated to dryness and redissolved in 3 mL of 60% methyl alcohol. Finally, the solutions were exposed to a UV light at a wavelength of 254 nm to measure enzymatic activity.

### Diamine and PA oxidase activity assay

PAO and DAO activities were determined by measuring the generation of H_2_O_2_, a PA oxidation product, according to the procedure of Su et al. (2005), with some modifications. Fresh samples were homogenized in 100 mM potassium phosphate buffer (pH 6.5). Then, the homogenates were centrifuged at 10,000 × g for 20 min at 4°C. The supernatants were used for the enzyme assay. Reaction mixtures contained 25 mL of potassium phosphate buffer (100 mM, pH 6.5), 0.2 mL of 4-aminoantipyrine/N,N-dimethylaniline reaction solutions, 0.1 mL of horseradish peroxidase (250 units mL^−1^), and 0.2 mL of enzyme extract. The reactions were initiated by adding 15 μL of 20 mM Put to analyze DAO determination and 20 mM Spd+Spm to analyze PAO. One unit of enzyme activity was defined as 0.001 absorbance units of the change in the optical density.

## Statistical analysis

Growth measurements were performed with 10 replicates. The results are expressed as the mean ± standard error (SE). One-way analysis of variance (ANOVA) combined with an LSD test was used to determine the significance of the differences between treatments. Structural equation modeling (SEM) was used to explain the direct effects of related components and PA types on PA metabolism according to Grace (2006). Each arrow represents a causal relationship, i.e., a change in the variable at the tail of an arrow is a direct cause of the change in the variable at the head. Nonsignificant paths are indicated by dotted arrows. Larger standardized coefficients (listed beside each significant path) indicate that the variable at the tail has a stronger effect on the variable at the head. The original SEM was based on the complete theoretical knowledge. The *X*^2^-test was used to determine whether covariance structures suggested by the model adequately fit the actual covariance structures of the data. A nonsignificant *X*^2^-test (*P* > 0.05) indicates adequate model fit. The model modification indices provide a strong tool for data exploration and hypothesis generation if the initial model does not adequately fit.

## Results

### Plant growth

The root fresh weight (FW) decreased significantly in response to salinity stress in both zoysia cultivars. The addition of exogenous Spd alleviated salinity-mediated growth reduction to a certain extent. Losses in FW due to salt stress under the NaCl treatments were 23.4% in z081 and 17.8% in z057, respectively, indicating the higher salt tolerance of z057 (Table 2).

**Table 2:** **Fresh weight of Zoysiagrass grown under salt stress with or without treatment with Spd for 8 days**.

**Cultivar**	**Treatment**	**Root fresh weight (g/cm^2^)**
z057	Control	0.431 ± 0.007a
	Spd	0.434 ± 0.004a
	NaCl	0.354 ± 0.01c
	NaCl+Spd	0.409 ± 0.01b
z081	Control	0.397 ± 0.003a
	Spd	0.399 ± 0.002a
	NaCl	0.304 ± 0.004c
	NaCl+Spd	0.356 ± 0.006b

*The data represent the means ± SEs from three independent experiments. Values in a single column sharing the same letters are not significantly different (p < 0.05; Duncan's multiple range test)*.

### Free PA contents

Free PA levels showed a great difference in response to salinity stress with exogenous Spd application (Table 3). Spd treatment had almost no effect on free PAs in plants that were not exposed to salt stress. Free Put, Spd, and Spm exhibited similar trends in both cultivars, but certain differences were observed (Table 3). Free Put, Spd and Spm in the roots of z057 demonstrated an upward trend for 4 days after salt stress. In z081, free Spd, Spm, and Put levels increased for 6 days after salt stress, followed by a decline. However, the increase in PA levels was maintained longer in z057 than in z081. Exogenous Spd enhanced PA levels in both cultivars in response to salt stress except free Spd. The upward trend in free Put observed during salt stress was suppressed to a certain extent by the application of Spd, whereas the upward trends in free Spd and Spm upward were enhanced (Table 3).

**Table 3:** **Effects of Spd, salt, and salt+Spd on levels of free Put, Spd, and Spm in roots of zoysia grass**.

**Species**	**Treatment**	**Free Put (nmol/g**^**−1**^ **FW)**					**Free Spd (nmol/g**^**−1**^ **FW)**					**Free Spm (nmol/g**^**−1**^ **FW)**				
	**Days**	**0**	**2**	**4**	**6**	**8**	**0**	**2**	**4**	**6**	**8**	**0**	**2**	**4**	**6**	**8**
z057	Control	630 ± 27	593 ± 23c	638 ± 23c	674 ± 33c	587 ± 17c	1,000 ± 29	1030 ± 62c	950 ± 19d	843 ± 35d	849 ± 25d	107 ± 5	114 ± 4c	96 ± 4c	90 ± 2c	107 ± 4c
	Salt	661 ± 21	1,537 ± 50a	2,262 ± 47a	1,837 ± 40a	1,507 ± 43a	1,068 ± 33	1,344 ± 31b	1,764 ± 44b	1,674 ± 30b	1,210 ± 45b	102 ± 4	199 ± 6b	242 ± 6b	198 ± 3b	137 ± 4b
	Spd	612 ± 11	582 ± 35c	594 ± 15c	621 ± 17c	637 ± 13c	1018 ± 34	1,286 ± 57b	1216 ± 61c	1172 ± 44c	672 ± 10c	104 ± 11	116 ± 8c	105 ± 4c	99 ± 3c	95 ± 2c
	Salt+Spd	642 ± 20	1,350 ± 32b	1,676 ± 38b	1670 ± 46b	949 ± 28b	1,035 ± 88	2,923 ± 73a	2,800 ± 58a	3,035 ± 116a	1,400 ± 36a	107 ± 9	302 ± 5a	419 ± 8a	300 ± 6a	256 ± 8a
	*p*	n.s.	^**^	^**^	^**^	^**^	n.s.	^**^	^**^	^**^	^**^	n.s.	^**^	^**^	^**^	^**^
z081	Control	750 ± 23	643 ± 22c	774 ± 11c	938 ± 37c	677 ± 11c	1,086 ± 41	1,053 ± 45c	946 ± 10d	1,112 ± 34c	962 ± 13c	95 ± 2	103 ± 7	91 ± 3	88 ± 3	94 ± 3
	Salt	749 ± 22	1,230 ± 50a	1,511 ± 57a	1,512 ± 57a	889 ± 27b	1,064 ± 52	1,314 ± 28b	1,645 ± 39b	1,700 ± 44b	1,464 ± 26a	94 ± 2	239 ± 5	197 ± 6	272 ± 9	145 ± 4
	Spd	758 ± 23	589 ± 12c	700 ± 17c	850 ± 40c	663 ± 15c	1,053 ± 58	1,065 ± 36c	1,130 ± 37c	1,100 ± 62c	1,150 ± 34b	104 ± 3	108 ± 4	97 ± 4	110 ± 4	95 ± 2
	Salt+Spd	749 ± 8	1,230 ± 58b	1,224 ± 62b	1,118 ± 29b	1,071 ± 38a	1,088 ± 17	1,668 ± 33a	1,833 ± 46a	2237 ± 56a	1,401 ± 58a	107 ± 3	228 ± 6	326 ± 6	302 ± 7	273 ± 5
	*p*	n.s.	^**^	^**^	^**^	^**^	n.s.	^**^	^**^	^**^	^**^	n.s.	^**^	^**^	^**^	^**^

The values represent the means ± SEs of three replications per treatment. Different letters indicate significant differences (P < 0.05) with respect to treatment for each species. n.s., not significant;

** *P < 0.05 indicates the significance of the main effects determined by ANOVA. The values in the same column followed by the same letter are not significantly different at P < 0.05*.

### Soluble conjugated PA contents

Soluble conjugated PA levels are reliable indexes of salt tolerance. In this study, we observed that only exogenous Spd application slightly reduced instead of enhanced conjugated PA levels under normal conditions (Table 4). Soluble conjugated PA contents increased under salt stress in both species but decreased as time progressed. However, the peaks occurred at different times in the two cultivars (Table 4). Exogenous Spd application enhanced PA levels to different extents under salt stress in both species, and root PA levels were higher in z081 than in z057 (Table 4).

**Table 4:** **Effects of Spd, salt, and salt+Spd on levels of soluble conjugated Put, Spd, and Spm in roots of zoysia grass**.

**Species**	**Treatment**	**Soluble conjugated Put (nmol/g**^**−1**^ **FW)**					**Soluble conjugated Spd (nmol/g**^**−1**^ **FW)**					**Soluble conjugated Spm (nmol/g**^**−1**^ **FW)**				
	**Days**	**0**	**2**	**4**	**6**	**8**	**0**	**2**	**4**	**6**	**8**	**0**	**2**	**4**	**6**	**8**
z057	Salt	372 ± 7	1,152 ± 39a	1,762 ± 83b	1,429 ± 53a	840 ± 43b	871 ± 32	2,341 ± 61b	2364 ± 53b	1,747 ± 78b	2,151 ± 40b	61 ± 2.1	73 ± 4.6b	135 ± 3.0b	139 ± 3.8b	83 ± 3.0b
	Spd	369 ± 5	357 ± 7b	356 ± 7c	498 ± 22b	508 ± 14c	851 ± 21	869 ± 15c	733 ± 12c	1116 ± 71c	734 ± 11c	62 ± 2.6	61 ± 2.1b	59 ± 1.2c	73 ± 1.5c	67 ± 3.0c
	Salt+Spd	383 ± 8	1,137 ± 49a	1,853 ± 72a	1,505 ± 47a	1,205 ± 39a	852 ± 26	2,782 ± 50a	3,196 ± 73a	2,504 ± 91a	2,487 ± 55a	63 ± 1.2	108 ± 4.2a	288 ± 6.7a	268 ± 5.2a	148 ± 3.8a
	*p*	n.s.	^**^	^**^	^**^	^**^	n.s.	^**^	^**^	^**^	^**^	n.s.	^**^	^**^	^**^	^**^
z081	Control	443 ± 10	474 ± 21bc	497 ± 12b	377 ± 5c	489 ± 14b	1419 ± 51	1,396 ± 31	1,594 ± 41d	1303 ± 31c	1,557 ± 39d	155 ± 3.8	62 ± 2.1d	69 ± 2.3c	40 ± 1.5c	67 ± 2.1d
	Salt	446 ± 6	516 ± 14ab	912 ± 15a	733 ± 6b	612 ± 11a	1,443 ± 39	3,101 ± 51	2,992 ± 55b	1,407 ± 43c	1,811 ± 51c	157 ± 2.6	177 ± 3.5b	283 ± 4.9a	88 ± 2.1b	96 ± 2.5b
	Spd	456 ± 23	459 ± 8c	500 ± 11b	336 ± 6d	479 ± 8b	1553 ± 172	2,333 ± 40	1,928 ± 40c	2,266 ± 48b	2,172 ± 60b	162 ± 9.7	118 ± 1.5c	101 ± 2.6b	37 ± 1.2c	79 ± 3.1c
	Salt+Spd	481 ± 8	545 ± 10a	897 ± 10a	965 ± 16a	600 ± 11a	1,550 ± 147	3,837 ± 37	3,334 ± 62a	2,451 ± 46a	3,250 ± 63a	159 ± 6.5	188 ± 2.6a	282 ± 4.6a	135 ± 3.1a	144 ± 4.2a
	*p*	n.s.	^**^	^**^	^**^	^**^	n.s.	^**^	^**^	^**^	^**^	n.s.	^**^	^**^	^**^	^**^

The values represent the means ± SEs of three replications per treatment. Different letters indicate significant differences (P < 0.05) with respect to treatment for each species. n.s., not significant;

** *P < 0.05 indicates the significance of the main effects determined by ANOVA. The values in the same column followed by the same letter are not significantly different at P < 0.05*.

### Insoluble bound PA contents

As molecules involved in osmotic adjustment, the content of insoluble bound PAs is lower than those of other PA forms. However, many studies have indicated that insoluble bound PAs are important for plant salt tolerance. Exogenous Spd influenced insoluble bound PAs slightly under normal conditions (Table 5). Insoluble bound PAs showed a similar tendency as soluble conjugated PAs under salt stress. Exogenous Spd application increased PA levels significantly at different times in both cultivars exposed to salinity stress (Table 5).

**Table 5:** **Effects of Spd, salt, and salt+Spd on levels of insoluble bound Put, Spd, and Spm in roots of zoysia grass**.

**Species**	**Treatment**	**Insoluble bound Put (nmol/g**^**−1**^ **FW)**					**Insoluble bound Spd (nmol/g**^**−1**^ **FW)**					**Insoluble bound Spm (nmol/g**^**−1**^ **FW)**				
	**Days**	**0**	**2**	**4**	**6**	**8**	**0**	**2**	**4**	**6**	**8**	**0**	**2**	**4**	**6**	**8**
z057	Control	113 ± 2.6	164 ± 4.0c	117 ± 3.1d	110 ± 3.2c	111 ± 2.6d	111 ± 5.3	129 ± 6.0c	107 ± 2.0d	123 ± 4.0b	107 ± 3.8c	35 ± 1.2	34 ± 1.5c	36 ± 1.5c	35 ± 3.0c	35 ± 1.2d
	Salt	111 ± 2.6	354 ± 6.9b	357 ± 7.7b	259 ± 6.1b	257 ± 4.6b	117 ± 2.8	642 ± 4.6a	1,168 ± 38a	760 ± 26a	736 ± 7.5a	34 ± 1.0	96 ± 2.5b	72 ± 2.3b	52 ± 2.1b	55 ± 2.1b
	Spd	116 ± 5.3	148 ± 2.6c	142 ± 1.7c	112 ± 2.1c	143 ± 3.1c	106 ± 5.5	123 ± 1.5c	140 ± 4.7c	133 ± 2.5c	114 ± 2.0d	36 ± 1.5	35 ± 1.5c	38 ± 0.6c	39 ± 1.0c	41 ± 1.0c
	Salt+Spd	119 ± 8.1	573 ± 9.6a	576 ± 8.5a	396 ± 7.5a	368 ± 4.7a	108 ± 6.1	533 ± 6.6b	688 ± 16b	731 ± 14a	663 ± 12.5b	35 ± 1.5	159 ± 7.0a	87 ± 3.2a	70 ± 1.5a	66 ± 1.7a
	*p*	n.s.	^**^	^**^	^**^	^**^	n.s.	^**^	^**^	^**^	^**^	n.s.	^**^	^**^	^**^	^**^
z081	Control	101 ± 2.6	115 ± 3.1c	100 ± 1.5c	120 ± 3.5c	97 ± 2.6c	103 ± 2.5	92 ± 2.5d	103 ± 3.1c	137 ± 2.6c	87 ± 3.8c	31 ± 1.0	31 ± 1.2d	32 ± 2.1d	43 ± 1.5d	52 ± 1.5c
	Salt	101 ± 2.5	269 ± 6.4b	357 ± 6.4b	341 ± 5.0b	244 ± 4.6b	102 ± 3.8	490 ± 11b	1,887 ± 59a	392 ± 11b	672 ± 8.6a	31 ± 0.6	97 ± 2.3b	85 ± 2.1b	75 ± 2.1b	65 ± 2.1b
	Spd	97 ± 3.2	106 ± 3.2c	97 ± 2.0c	120 ± 3.0c	98 ± 2.6c	115 ± 3.1	119 ± 3.2c	101 ± 2.1c	143 ± 4.0c	108 ± 2.6c	32 ± 1.5	70 ± 3.0c	65 ± 3.1c	65 ± 2.6c	54 ± 2.0c
	Salt+Spd	102 ± 2.1	363 ± 5.6a	478 ± 8.0a	547 ± 8.0a	411 ± 7.6a	104 ± 2.6	549 ± 6.8a	951 ± 19b	645 ± 9.0a	599 ± 9.5b	33 ± 1.0	166 ± 4.0a	98 ± 1.0a	89 ± 2.3a	75 ± 3.1a
	*p*	n.s.	^**^	^**^	^**^	^**^	n.s.	^**^	^**^	^**^	^**^	n.s.	^**^	^**^	^**^	^**^

The values represent the means ± SEs of three replications per treatment. Different letters indicate significant differences (P < 0.05) with respect to treatment for each species. n.s., not significant;

** *P < 0.05 indicates the significance of the main effects determined by ANOVA. The values in the same column followed by the same letter are not significantly different at P < 0.05*.

### PA biosynthetic enzyme activity

**Figure 1:**
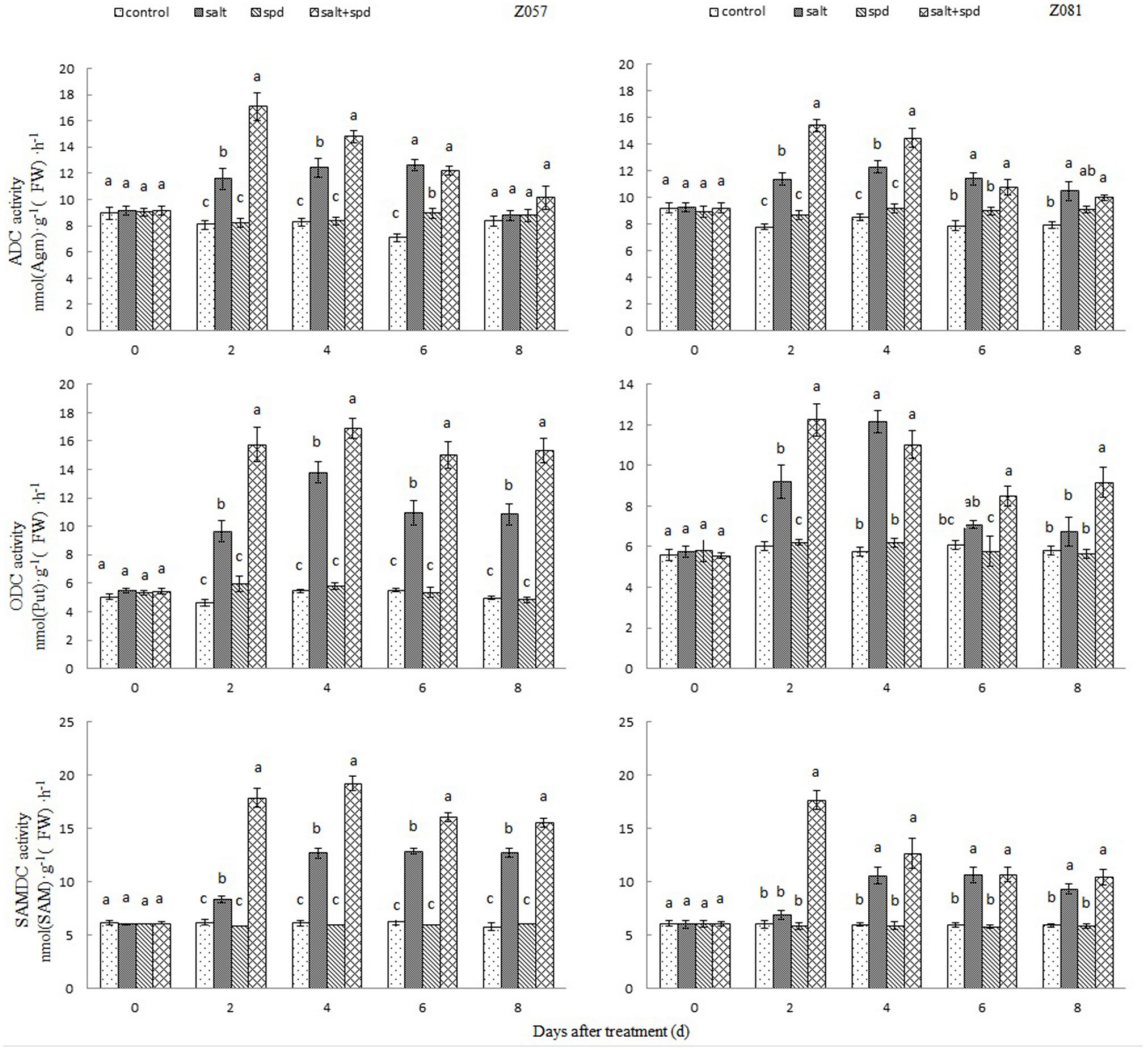
**Effects of Spd, salt and salt+Spd on activities of ADC, ODC, and SAMDC in roots of zoysia grass under 150 mM NaCl stress**. The data represent the means ± SEs of three replicates. Values in a single column sharing the same letters were not significant difference (*p* < 0.05) (Duncan's multiple range tests).

ODC activity in the roots of both cultivars increased after 2 days of salinity and peaked on day 4 (Figure 1). The increase in ODC activity was elevated by the application of exogenous Spd during salt stress. ODC activity levels induced by exogenous Spd were greater in z057 than in z081 (Supplementary Table S2). ADC activity in the roots increased rapidly after 2 days and peaked at 4 and 6 days in z081 and z057 under saline conditions, respectively (Supplementary Figure S1). Furthermore, very high levels of ODC and ADC activity were maintained in the Spd+salt-treatment (Supplementary Figure S1). Exogenous Spd had almost no effects on the two cultivars under normal conditions except ADC activity on day 6 in z057 (Supplementary Table S1).

Salt stress caused a significant increase in SAMDC activity in the roots of both cultivars (Figure 1). This increase in SAMDC activity was enhanced and peaked on day 4 under salt stress, although the activity in z057 was higher than that in z081. Exogenous Spd led to higher and more persistent levels of SAMDC activity in z057 than in z081 under salt stress. In both cultivars, exogenous Spd had almost no effects on PA biosynthetic enzyme activity under nonsaline conditions (Figure 1).

### PA degradative enzyme activity

**Figure 2:**
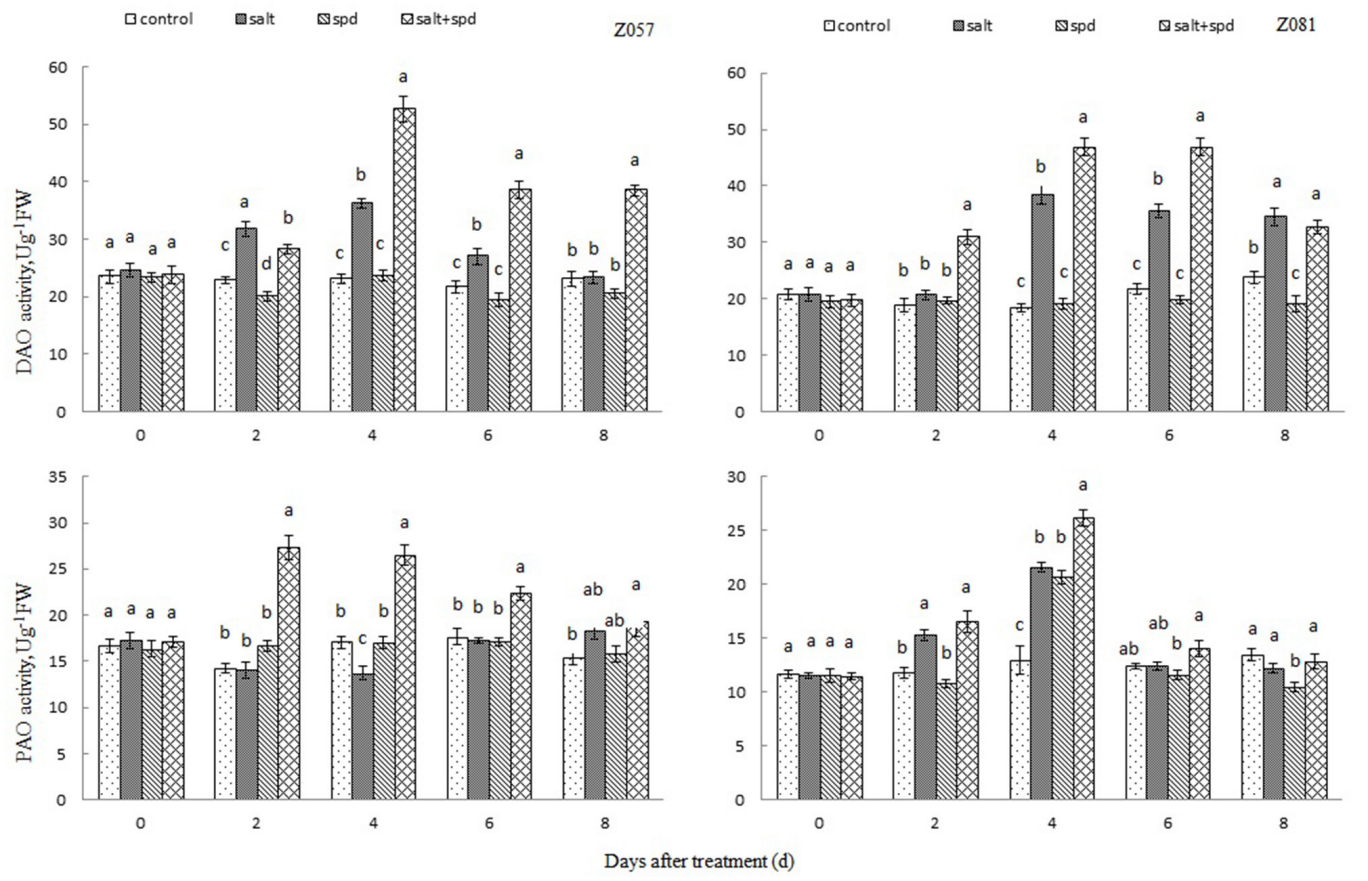
**Effects of Spd, salt and salt+Spd on activities of DAO and PAO in roots of zoysia grass under 150 mM NaCl stress**. The data represent the means ± SEs of three replicates. Values in a single column sharing the same letters were not significant difference (*p* < 0.05) (Duncan's multiple range tests).

During salt stress, PAO activity increased rapidly in both cultivars (Figure 2). However, it decreased rapidly in z057 and gradually in z081. Exogenous Spd led to higher PAO activity in the roots of z081 under salt stress.

Salt stress induced a rapid increase in DAO activity in z081 roots but had little effect on z057 roots. Exogenous Spd enhanced DAO activity in both cultivars under salt stress (Figure 2).

### Pathway analysis of polyamine metabolism

**Figure 3:**
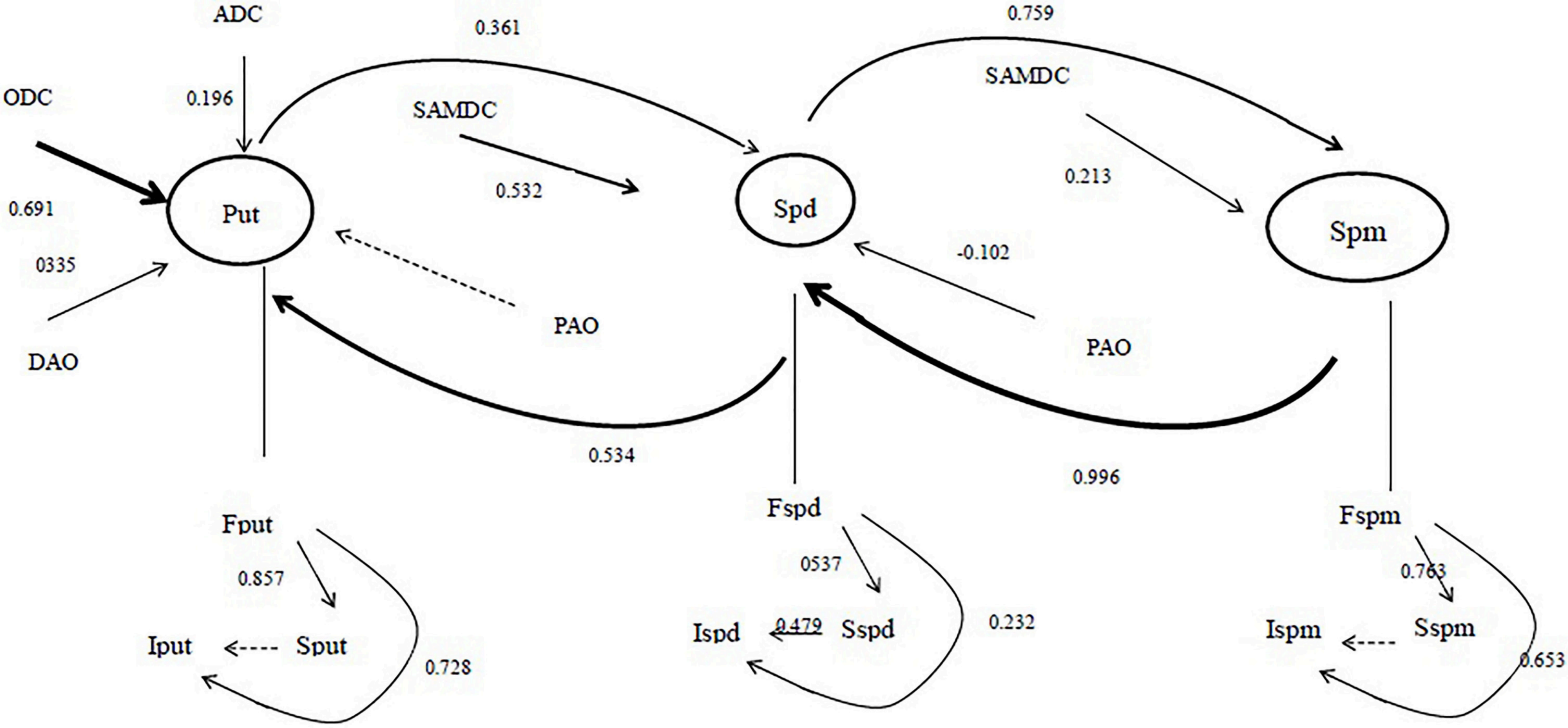
**The structural equation model linking ***Zoysia japonica*** Steud. polyamine metabolism to related components**. Each arrow represents a causal relationship, i.e., a change in the variable at the tail of an arrow is a direct cause of the change in the variable at the head. Nonsignificant paths are indicated by dotted arrows. Larger standardized coefficients (listed beside each significant path) indicate that the variable at the tail has a stronger effect on the variable at the head. F, free; S, soluble conjugated; I, insoluble bound; Put, diamine putrescine (nmol/g^−1^ FW); Spd, triamine spermidine (nmol g^−1^ FW); Spm, tetraamine spermine (nmol g^−1^ FW); ADC, arginine decarboxylase (nmol(Agm)·g^−1^(FW)·h^−1^); ODC, ornithine decarboxylase (nmol(Put)·g^−1^(FW)·h^−1^); SAMDC, S-adenosylmethionine decarboxylase (nmol(SAM)·g^−1^(FW)·h^−1^); PAO, polyamine oxidase (U g^−1^ FW); DAO, diamine oxidase (U g^−1^ FW).

SEM was used to explain the direct effects of related components and PA types on PA metabolism. The original SEM was based on the complete theoretical knowledge. SEM showed that ODC was the main enzyme with a direct effect (0.691) on Put synthesis, and DAO showed direct effects (0.335) on Put catabolism (Figure 3). In addition, SAMDC and the endogenous Spd level showed direct effects of 0.532 and 0.213 on endogenous Spd and Spm levels, respectively. Furthermore, the endogenous Spm and Spm levels also affected the endogenous Spd and Put levels, respectively. As for the different PA forms, the free forms showed direct effects on the soluble conjugated forms among the major PAs (Figure 3).

## Discussion

Salt stress involves a combination of osmotic stress and dehydration due to excess sodium ions and adversely limits plant growth and development (Rossetto et al., 2015). It has been widely reported that PA metabolism is one of the defense mechanisms that plants invoke in response to salt stress (Kusano et al., 2008). In this study, salt stress reduced the zoysia FW, and exogenous Spd application protected the FW from salt-induced injury (Table 1). This study found a similar pattern of results as previous research that exogenous PAs reduced the decline in plant FW upon exposure to salt stress (Hu et al., 2012).

Many studies have reported changes in PA levels under salt stress (Marco et al., 2011). PA contents differ following short-term and long-term exposure to salinity. Hu et al. (2012) reported the disturbance of PA homeostasis under short-term salt stress in tomato roots. In general, high Spd and Spm values are considered salt tolerance indices, as demonstrated by Li et al. (2016) in salt stress-sensitive and salt stress-resistant zoysiagrass varieties. In a previous study, PA levels changed under salt stress: Put levels decreased and Spd and Spm levels increased in all species examined (Zapata et al., 2004). In other species, an increase in the (Spd+Spm)/Put ratio was observed under salt stress, and Spd and Spm contributed to osmotic stress tolerance in wheat seedlings (Liu et al., 2004). In addition, the concentrations of different PA forms (free, soluble, and insoluble) differ greatly under salt stress conditions with exogenous Spd application (Jia et al., 2010; Hu et al., 2012). In the present study, we determined the variation of the dynamic levels of different PA forms exposed to different conditions. Our results indicated that the three PA forms increased in the first stage and then decreased with increasing time following exposure to salt stress. Exogenous Spd application enhanced the levels of all PAs except free Put (Table 3). These data suggested that exogenous Spd might improve zoysia growth and play a role in the regulation of PA forms in response to salinity stress.

ADC, ODC, and SAMDC activities change during environmental stress tolerance in most plant species, indicating that these enzymes are regulated by PA metabolism (Bagni and Tassoni, 2001; Liu et al., 2007). Diamine Put is synthesized by ADC or ODC, and triamine Spd is synthesized by SPDS from Put via the addition of an aminopropyl moiety donated by decarboxylated S-adenosylmethionine (dcSAM) formed by SAMDC (Hanfrey et al., 2002). The elevated activities of ADC, ODC, and SAMDC were a response to the enhancement of PA levels. In the present study, ODC and SAMDC activity were increased in both cultivars exposed to salt stress, and the pattern of change was consistent with the levels of certain PAs (Tables 3–5 and Figures 1, 2). Furthermore, exogenous Spd enhanced ADC activity, ODC activity as well as SAMDC activity in both cultivars (Figure 3).

DAO, which is localized to the plant cell wall, facilitates Put catabolism and is important for cross-linking reactions under stress conditions (Eller et al., 2006). Exogenous Spd increased DAO activity due to the concomitant decrease in free Put content. The application of exogenous Spd induced significant increases in Spd and Spm contents, which were attributable to increased SAMDC activity. Despite the large increases in ADC and ODC activities in both cultivars, little free Put accumulated in the roots due to the large increase in DAO and PAO activity and the conversion of free Put to conjugated and bound Put and free Spd and Spm (Ndayiragije and Lutts, 2006).

PA metabolism is a very complex multistep process that is affected by many factors. PA levels are a quantitative characteristic of the salt-stress response. The effect of enzymes on Put levels depends on the activities of ODC and DAO, which are involved in Put catabolism (Figure 3). The SEM indicated that the activity of SAMDC contributed to Spd and Spm levels, whereas endogenous Spd contributed more to Spm than SAMDC activity (Figure 3). Many studies have reported that the three major PAs are present in three forms, and the three forms all showed dynamic changes to different extents under salt stress conditions with exogenous Spd application. Our current results indicate that the level of free PAs contributed to soluble and insoluble PA level among the major PAs, whereas soluble PAs (put and Spm) did not contribute to the levels of the corresponding PA forms (Figure 3).

In summary, salt stress decreased the zoysiagrass FW and increased free Spd and Spm and soluble and insoluble Put, Spd and Spm levels in both cultivars. Moreover, salt stress enhanced the activity of ODC, SAMDC, and DAO. Exogenous Spd improved PA metabolism in response to salt stress. In addition, ODC, SAMDC, and DAO are the main enzymes of PA metabolism, and endogenous Spd levels also for endogenous Spm levels. Free PA forms may be the primary factor influencing the variations of other PA forms.

## Author contributions

SL, PM, YW designed research; SL, PM, YW performed research; SL, PM, YW, YZ contributed new reagents/analytic tools; SL and LC analyzed data; and SL and YZ wrote the paper.

### Conflict of interest statement

The authors declare that the research was conducted in the absence of any commercial or financial relationships that could be construed as a potential conflict of interest.
