# Supplementary material for: The Variation Tendency of Polyamines Forms and Components of Polyamine Metabolism in Zoysiagrass (Zoysia japonica Steud.) to Salt Stress with Exogenous Spermidine Application
Source: Front Physiol. 2017 Apr 6;8:208. doi: 10.3389/fphys.2017.00208 (PMC5382195; doi:10.3389/fphys.2017.00208)
Supplement: Supplementary file 1 [file Table1.docx]

Table S1. Effects of Spd, salt and salt+Spd on activities of ADC in roots of zoysia grass under 150 mM NaCl stress (P < 0.05).

| Cultivar | Treatment | ADC (nmol(Agm)·g^-1^( FW) ·h^-1^ ) | | | | |
| --- | --- | --- | --- | --- | --- | --- |
|  |  | 0d | 2d | 4d | 6d | 8d |
| Z057 | Control | 8.95 | 8.06 | 8.27 | 7.11 | 8.33 |
|  | Salt | 9.15 | 11.54 | 12.40 | 12.63 | 8.78 |
|  | Spd | 9.03 | 8.21 | 8.38 | 8.94 | 8.76 |
|  | Salt +Spd | 9.16 | 17.10 | 14.78 | 12.18 | 10.15 |
| Z081 | Control | 9.23 | 7.81 | 8.50 | 7.89 | 7.93 |
|  | Salt | 9.26 | 11.40 | 12.30 | 11.43 | 10.50 |
|  | Spd | 8.93 | 8.69 | 9.18 | 9.00 | 9.13 |
|  | Salt +Spd | 9.23 | 15.43 | 14.49 | 10.75 | 10.01 |

Table S2. Effects of Spd, salt and salt+Spd on activities of ODC in roots of zoysia grass under 150 mM NaCl stress (P < 0.05).

| Cultivar | Treatment | ODC (nmol(Put)·g^-1^( FW) ·h^-1^) | | | | |
| --- | --- | --- | --- | --- | --- | --- |
|  |  | 0d | 2d | 4d | 6d | 8d |
| Z057 | Control | 5.24 | 4.61 | 5.46 | 5.53 | 4.95 |
|  | Salt | 5.36 | 9.66 | 13.79 | 10.96 | 10.87 |
|  | Spd | 5.31 | 5.95 | 5.83 | 5.37 | 4.83 |
|  | Salt + Spd | 5.33 | 15.76 | 16.88 | 15.02 | 15.36 |
| Z081 | Control | 5.59 | 6.03 | 5.76 | 6.08 | 5.82 |
|  | Salt | 5.77 | 9.19 | 12.16 | 7.09 | 6.76 |
|  | Spd | 5.80 | 6.21 | 6.18 | 8.89 | 5.64 |
|  | Salt + Spd | 5.55 | 12.26 | 11.04 | 8.49 | 9.17 |

Figure S1. Effects of Spd, salt and salt+Spd on activities of ADC and ODC in roots of zoysia grass under 150 mM NaCl stress. The data represent the means ± SEs of three replicates. Values in a single column sharing the same letters were not significant difference (p < 0.05) (Duncan’s multiple range tests).
